# Commentary: Renal Function Estimation and Cockcroft–Gault Formulas for Predicting Cardiovascular Mortality in Population-Based, Cardiovascular Risk, Heart Failure and Post-Myocardial Infarction Cohorts: The Heart ‘OMics’ in AGEing (HOMAGE) and the High-Risk Myocardial Infarction Database Initiatives

**DOI:** 10.3389/fmed.2017.00077

**Published:** 2017-06-12

**Authors:** Thomas J. Wilkinson, Douglas W. Gould, Emma L. Watson, Alice C. Smith

**Affiliations:** ^1^Leicester Kidney Exercise Team, Department of Infection, Immunity and Inflammation, University of Leicester, Leicester, United Kingdom; ^2^John Walls Renal Unit, University Hospitals of Leicester Trust, Leicester, United Kingdom

**Keywords:** renal function, kidney, nephrology, muscle, exercise, nutrition

In a recent study, Ferreira and colleagues (1) describe the association of common equations to estimate renal function *via* “estimated glomerular filtration rate” (eGFR) with mortality. The authors suggest that while the Cockcroft–Gault-body surface area (BSA) method is more accurate in predicting cardiovascular mortality in patients with existing cardiovascular risk, the CKD-EPI (Chronic Kidney Disease Epidemiology Collaboration equation) provides the best compromise between renal function and cardiovascular mortality prediction. As the authors state, the measurement of accurate renal function is important in the diagnosis and treatment of chronic kidney disease (CKD), adjustment of drug doses, and decision-making regarding renal replacement therapy initiation (1, 2).

In all methods described by Ferreira et al. (1), eGFR measurement is based on serum creatinine clearance. Creatinine is a 113-kDa breakdown product of muscle metabolism (3) and is the most commonly used filtration marker and indicator of renal function. However, its measurement suffers from a variety of analytical interferences and standardization problems (2). Such non-GFR determinants include age, sex, and ethnicity. Muscle mass and dietary protein intake can also affect creatinine concentrations; both these factors are often concomitant with exercise levels. In a population where muscle wasting and inadequate physical activity and protein intake is problematic, we feel it is important to highlight the critique of creatinine-based eGFR in CKD, and how creatinine can be influenced by difference factors, principally muscle mass, and exercise. We also examine the role of cystatin C (CysC) and its role as a confirmatory biomarker in these patients.

## Muscle Mass

As formation occurs almost exclusively in the muscle, creatinine excretion is influenced by muscle mass (2). As such, reductions in muscle are associated with reduced creatinine clearance, resulting in an overestimated eGFR. Conversely, higher muscle mass is associated with increased creatinine and underestimated eGFR. Muscle mass (and therefore creatinine concentration) is extremely variable among the elderly and children (2), and particularly in CKD patients experiencing muscle wasting (4). As such, eGFR based on creatinine may not be the most accurate renal function measure in patients with such excesses of muscle mass. Notably, the National Institute for Health and Care Excellence guidelines (5) recommend that in patients with “extreme” body composition (“those with muscle wasting disorders”) “caution” should be taken when interpreting eGFR by creatinine. It is important to state that the different equations (1) often used to predict GFR do not include body composition parameters.

Due to the inclusion of body mass in the formula, both the Cockcroft–Gault method and modification of diet in renal disease (MDRD) equation (1) are particularly erroneous in patients with poor or high muscle mass (2). In particular, the MDRD equation is known to overestimate GFR in older populations, possibly as a result of reduced muscle mass (and thus lower creatinine concentration) (6). In individuals with high creatinine production and muscle mass, underestimation of MDRD eGFR is expected.

## Exercise

Bouts of exercise can also influence creatinine concentration as creatine is broken down from the muscle. Several studies have shown transient increases in creatinine concentration (and a subsequent drop in eGFR) following prolonged exercise events such as triathlons and endurance running/cycling events (7). Notably, creatinine-based eGFR was reduced by 16% immediately following a 21 km run (8), whereas creatinine-based eGFR was reduced by 30% following 30 min exercise at 80% VO_2_ peak (9). While the understanding of renal function following exercise is important in healthy individuals, its knowledge is essential in patients with existing renal complications. Early work (10) demonstrated that moderate exercise increased plasma creatinine by ~10% in patients with a kidney transplant, although exercise had no detrimental effect on renal function. Further research is needed to fully understand acute renal changes following exercise in CKD groups. Specifically, sustained elevated creatinine following exercise may result in false eGFR readings.

Increases in plasma creatinine concentration are possibly the result of release of creatinine from the working muscles, dehydration, or changes in renal hemodynamics (e.g., reduction in renal blood flow) (7, 9). Despite this temporary increase in plasma creatinine and reduction in eGFR, it is thought to be of little clinical relevance or concern to renal function. However, some investigations have observed small indices of renal damage following prolonged strenuous endurance exercise. Further evidence is needed to determine whether repeated performance of endurance events leads to clinically concerning renal alterations (7).

## Dietary Protein Intake

Intake of protein rich in the amino acids arginine and glycine, precursors of creatine and guanidoacetate production, can increase creatinine concentrations, as can direct creatinine ingestion itself. Ingestion of creatine (either *via* red meat or supplementation) can increase muscle creatine “pools,” raising the urinary excretion of creatinine following creatine breakdown (2).

## CysC—A More Robust Measure?

We have described how creatinine–eGFR can be influenced by muscle mass, protein intake, and exercise: all factors affecting renal patients. CysC, a 113-kDa serum protein that is filtered and metabolized after tubular reabsorption, is a possible endogenous filtration marker that could be used to obtain a more accurate measure of kidney function. CysC is produced by all nucleated cells and is seemingly unaltered in inflammatory conditions (2). It is also less unaffected by age, sex (2, 3), weight, and muscle mass (2, 11) (although only after adjustment for BSA) (12).

Nevertheless, non-GFR determinants are less well known for CysC, and creatinine-derived eGFR remains the best measure in *routine* clinical practice. Despite recent advancements, CycC assays currently lack the same agreement and cost-effectiveness as creatinine-based ones and remain only a confirmatory test in practice. It has been demonstrated that simultaneous CysC and creatinine estimates are more accurate (3), therefore, due to the effect of exercise, diet, and muscle mass, in individuals perhaps whom do exercise with higher levels of physical activity, muscle mass, and protein intake, supplementary testing is required [e.g., addition of concurrent CysC testing or “gold standard” 24 h urine testing (9)]. An accurate measure of GFR, combined with a better understanding of eGFR determinants following exercise, will promote its safety and help avoid unnecessary avoidance of physical activity in renal patients.

## Author Contributions

Please see below author contributions in line with the ICMJE [(1) substantial contributions to conception and design, acquisition of data, or analysis and interpretation of data; (2) drafting the article or revising it critically for important intellectual content; and (3) final approval of the version to be published. Authors should meet conditions (1), (2), and (3)]: TW: (1) substantial contributions to conception and design, (2) drafting the article, (3). DG: (2) critically for important intellectual content, (3). EW: (2) critically for important intellectual content, (3). AS: (1) substantial contributions to conception and design, (2) critically for important intellectual content, (3).

## Conflict of Interest Statement

The authors declare that the research was conducted in the absence of any commercial or financial relationships that could be construed as a potential conflict of interest.
